# Effects of Replacing Extruded Maize by Dried Citrus Pulp in a Mixed Diet on Ruminal Fermentation, Methane Production, and Microbial Populations in Rusitec Fermenters

**DOI:** 10.3390/ani10081316

**Published:** 2020-07-30

**Authors:** Jairo García-Rodríguez, Cristina Saro, Iván Mateos, Jesús S. González, María Dolores Carro, María José Ranilla

**Affiliations:** 1 Departamento de Producción Animal, Universidad de León, 24007 León, Spain; jgarr@unileon.es (J.G.-R.); csarh@unileon.es (C.S.); imata@unileon.es (I.M.); jsgona@unileon.es (J.S.G.); 2 Instituto de Ganadería de Montaña, CSIC-Universidad de León, Finca Marzanas s/n, 24346 Grulleros, Spain; 3 Departamento de Producción Agraria, Escuela Técnica Superior de Ingeniería Agronómica, Agroalimentaria y de Biosistemas, Universidad Politécnica de Madrid, Ciudad Universitaria, 28040 Madrid, Spain; mariadolores.carro@upm.es

**Keywords:** citrus pulp, extruded maize, qPCR, ARISA, microbial protein synthesis, methane, Rusitec

## Abstract

**Simple Summary:**

Citrus pulp is the main by-product obtained from citrus processing. The high-moisture content of this by-product makes it rapidly perishable, its accumulation can cause environmental problems, and it causes high disposal costs for citrus processing factories. Therefore, alternative uses for citrus pulp are necessary, its use in ruminant feeding being one of the most feasible ones. In this study, we assessed the effects of replacing extruded maize in a diet for dairy sheep (20% of diet) by dried citrus pulp using an in vitro technique (Rusitec fermenters). Results showed some positive effects of citrus pulp on diet degradability and in vitro fermentation parameters. The growth of ruminal microbes and bacterial diversity were essentially unaffected. Our results indicate that maize in dairy sheep diets can be totally replaced by dried citrus pulp without negatively affecting ruminal fermentation. The use of citrus pulp would reduce the amount of human-edible ingredients used in the diet of dairy sheep.

**Abstract:**

Citrus pulp is a highly abundant by-product of the citrus industry. The aim of this study was to assess the effects of replacing extruded maize (EM; 20% of total diet) by dried citrus pulp (DCP; 20%) in a mixed diet on rumen fermentation and microbial populations in Rusitec fermenters. The two diets contained 50% alfalfa hay and 50% concentrate, and the same protein level. Four Rusitec fermenters were used in a cross-over design with two 13-d incubation runs. After 7-d of diet adaptation, diet disappearance, fermentation parameters, microbial growth, and microbial populations were assessed. Fermenters receiving the DCP showed greater pH values and fiber disappearance (*p* < 0.001) and lower methane production (*p* = 0.03) than those fed EM. Replacing EM by DCP caused an increase in the proportions of propionate and butyrate (*p* < 0.001) and a decrease in acetate (*p* = 0.04). Microbial growth, bacterial diversity, and the quantity of bacteria and protozoa DNA were not affected by the diet, but the relative abundances of fungi and archaea were greater (*p* < 0.03) in solid and liquid phases of DCP fermenters, respectively. Results indicate that DCP can substitute EM, promoting a more efficient ruminal fermentation.

## 1. Introduction

The citrus fruits are one of the largest fruit crops in the world [1]. Although most citrus fruits are consumed fresh, around 20% of the total world production is processed to obtain juice according to FAO [2]. Some countries have developed an important citrus juice canning industry that generates high economic benefits, as well as large amounts of waste. In 2018, Spain was the sixth world citrus producer country and the first in the European Union (EU) [3], with a production of more than 7.5 million tons, and about 17% were used to obtain juice [4]. Citrus pulp is the whole solid waste obtained after squeezing citrus fruits for juice extraction, and it is the main by-product obtained from citrus processing. Citrus pulp is composed of different parts of the fruits—peels, membranes, seeds, and residual pulp—and it can represent between 49% and 69% of the fresh weight of the fruit processed [5]. This by-product deteriorates rapidly due to its high content in moisture and nutrients, and its decomposition releases pollutant sludge that causes environmental damage [5]. Moreover, the seasonal production of citrus fruits results in high amounts generated in a short period of time. The high-moisture content of citrus pulp causes high storage, transportation, and disposal costs for the juice companies [6], and thus alternative uses are necessary. One feasible alternative is its use in ruminant feeding, and not only citrus pulp but also the surplus of citrus production and unmarketable whole fruits have been used to feed ruminants in citrus-producing areas for many years [7,8]. However, preservation methods, such as drying, are necessary to routinely include this by-product in ruminant diets. The drying process usually involves shedding, liming, pressing, and drying the pulp [5] to obtain dried citrus pulp (DCP). Thus, drying can increase the shelf life of citrus by-products and their storage, transportation, and handling conditions.

The chemical composition of citrus pulp depends on several factors, such as soil and climate conditions, cultivation practices, ripeness, and species or varieties of the fresh citrus fruits [9]. The content in pectin and soluble sugars of DCP is usually high, whereas crude protein (CP; average 69 g/kg dry matter (DM); [5]) and lignin content is low, and that of neutral detergent fiber (NDF) and acid detergent fiber (ADF) is intermediate [5]. Due to the great content of highly-degradable NDF and readily fermentable carbohydrates, DCP has a high nutritional value and it has been proposed as a possible substitute of cereals in ruminant diets [10,11]. Several in vitro [12,13,14] and in vivo [15,16,17] studies evaluated the inclusion of DCP in the diet of small ruminants, mainly in sheep. These studies showed that replacing barley or other cereals by DCP can increase rumen pH [18,19], fiber digestibility [15,20], and acetate proportion [10,21,22], with no effects on either total volatile fatty acid (VFA) production [10,21] or microbial protein synthesis (MPS) [21]. Moreover, other studies reported no negative effects on animal performance [15,22,23] and the quality of animal products [17,24].

However, little is known on the possible effects of DCP on rumen microbial growth and microbial populations. Ariza et al. [21] observed that replacing hominy feed by DCP tended to increase the efficiency of microbial growth in continuous-culture fermenters. In contrast, Barrios-Urdaneta et al. [20] reported that replacing increasing amounts of barley by DCP in the diet of sheep caused a linear decrease in the ruminal concentrations of both total and cellulolytic bacteria. The aim of this study was to assess the effects of replacing extruded maize in a diet for dairy sheep by DCP on ruminal fermentation characteristics, methane production, MPS, microbial populations, and bacterial diversity in Rusitec fermenters. To the best of our knowledge, the present study is the first to evaluate the effects of replacing extruded maize by DCP on methane production and ruminal microbial populations.

## 2. Materials and Methods

Sheep management and rumen content withdrawal were carried out by skilled personnel in accordance with the Spanish guidelines for experimental animal protection (Royal Decree 53/2013, of 1 February, on the protection of animals used for experimentation or other scientific purposes). Experimental protocols were approved by the León University Institutional Animal Care and Use Committee (approval number ULE_014_2016).

### 2.1. Animals and Feeding

Four rumen-cannulated Merino sheep (54.2 ± 2.58 kg of body weight) were used as ruminal content donors to inoculate the Rusitec fermenters. Animals were fed a diet composed of 54% forage and 46% concentrate that was distributed in two equal meals at 09:00 and 18:00 for 4 weeks before starting the in vitro incubation. The diet was fed at a fixed rate of 42 g of DM per kg of body weigh^0,75^. Table 1 shows the ingredients and chemical composition of the diet fed to donor sheep.

### 2.2. Experimental Diets

Two diets composed of 50% alfalfa hay and 50% concentrate, with the concentrate composed by 20% of either extruded maize (EM diet) or DCP (DCP diet), were formulated. Diets were designed to be representative of those fed to dairy sheep, and to contain 16% of CP and more than 30% NDF. The ingredients and chemical composition of both diets are shown in Table 2. Alfalfa hay was chopped (about 0.5 cm pieces) and concentrate was ground through a 3 mm sieve before incubation in the fermenters. The extruded maize contained 994, 69, 72, and 30 g/kg DM of organic matter (OM), CP, NDF, and ADF, respectively. The DCP used in this study was a commercial-pelleted product consisting of orange and tangerine pulp, and contained 802, 54, 250, and 180 g/kg DM of OM, CP, NDF, and ADF, respectively.

### 2.3. Rusitec Trial

Four Rusitec fermenters (600 mL effective volume) were used in a cross-over design with two 13-day incubation periods each. The general incubation procedure was performed as described by Martinez et al. [25]. In each period, 2 fermenters received daily 30 g DM of the EM diet fed into nylon bags (100 µm pore size), whereas the other 2 fermenters received 30 g of the DCP diet. The first day of each incubation period, ruminal contents from sheep were obtained immediately before the morning feeding and were strained through four layers of cheesecloth into pre-warmed thermal flasks with an O_2_-free headspace. Solid contents were also preserved in a thermal-flask and both were transported to the laboratory where they were transferred to fermenters within 30 min of collection. Each fermenter was inoculated with 250 mL of strained rumen fluid, 200 mL of artificial saliva [26], and 80 g of solid rumen content supplied into a nylon bag. Each of the following days, a nylon bag containing the undigested diet after 48 h incubation was taken out the fermenters and was replaced by a nylon bag containing the diet. Artificial saliva was continuously infused into each fermenter at a rate of 665 mL/day (dilution rate 4.17%/h). The dilution rate and solids retention time were chosen to resemble values previously observed in vivo in sheep [27,28].

Each incubation period consisted of 7-d of diet adaptation, followed by a 6-d sampling period. From day 5 to 9, a solution of ^15^NH_4_Cl was added to the artificial saliva at a rate of 4.0 mg of ^15^N/g of dietary N to label the ruminal bacteria for measuring MPS. On days 8 and 9, liquid (LIQ) digesta (effluents) and solid (SOL) digesta from nylon bags were collected to determine MPS in both phases as described by Carro and Miller [29]. About 500 mL of effluent were used for liquid-associated bacteria isolation as described by Martinez et al. [25], and the rest of the effluent was freeze-dried for DM determination and ^15^N enrichment analysis. The contents of nylon bags were thoroughly mixed and used for DM determination, ^15^N enrichment analysis, and isolation of solid-associated bacteria as detailed by Martínez et al. [25]. Microbial populations were assessed in samples of both SOL and LIQ phase from fermenters.

On days 10, 11, 12, and 13, gas samples were taken for analysis of methane and samples from effluent were collected for VFA and NH_3_-N analyses. In addition, nylon bags were washed and dried to determine the diet apparent disappearance following the procedures described by Martinez et al. [25].

### 2.4. DNA Extraction, Automated Ribosomal Intergenic Spacer Analysis (ARISA), and Quantitative Polymerase Chain Reaction Analyses (qPCR)

Samples of the LIQ and SOL digesta were freeze-dried before extraction of DNA. The DNA was isolated in triplicate from 120 mg samples of SOL and LIQ digesta following the procedure described by Yu and Morrison [30] with an additional step involving the treatment of samples with cetyltrimethylammonium bromide for PCR inhibitors removal [31]. The QIAmp DNA Stool Mini Kit columns (QIAgen, Valencia, CA, USA) were used to purify the DNA. A Nanodrop ND-1000 (NanoDrop Technologies, Wilmington, DE, USA) was used to quantify the eluted DNA, and the evaluation of DNA purity was carried out by measuring the absorbance ratios (A260:A280 and A260:A230). Absorbance ratios were between 1.83 and 1.94 for A260:280, and between 1.74 and 2.25 for A260:A230, and the quality of the DNA was considered satisfactory.

The internal transcribe spacer of DNA was amplified using universal primers 16S-1392F and 23S-125R [32] for ARISA analysis, according to Saro et al. [33]. Thermocycling was conducted in a 2720 Thermal Cycler (Applied Biosystems, Foster City, CA, USA), and the automated detection of ARISA fragments was done in an ABI Prism 3130 Genetic Analyzer (Applied Biosystems, Foster City, CA, USA). Peaks were determined by comparison with an internal size standard using the GeneMaker Software v1.80 (SoftGenetics, State College, PA, USA). The presence or the absence of a peak were used to compare the profiles of the electropherograms by means of a dissimilarity matrix. The differences between groups of samples were assessed using a principal coordinate analysis (PCoA) based on the Bray–Curtis dissimilarities using the R environment and R package vegan [34].

Quantification of total bacteria and protozoa, as well as the relative quantification (determined in relation to the total bacterial population) of fungi and methanogenic archaea in LIQ and SOL digesta, were performed by qPCR. The qPCR was carried out in duplicate using an ABI PRISM 7000 Sequence Detection System (Applied Biosystems, Warrington, UK) as previously described by Saro et al. [31]. The DNA extracted from microbial pellets previously isolated from the rumen of sheep by our group was used as a standard for bacteria and protozoa, following the procedure described by Saro et al. [31]. The primers used to determine general bacteria and fungi were described by Denman and McSweeney [35], while those used for protozoa and methanogenic archaea have been described by Sylvester et al. [36] and Denman et al. [37], respectively. Amplification efficiencies for each primer pair were assessed by examining the dilution series (from 10^−1^ to 10^−5^) of a pooled DNA template in triplicate. Then, the observed threshold cycle (*C_T_*) values were plotted against the logarithm of total DNA concentration. Values of slopes (from −3.47 to −3.68) and regression coefficients (0.99) were similar to those previously reported for the same primers by Denman and McSweeney [35] for bacteria and fungi, and by Sylvester et al. [36] for protozoa. PCR efficiencies varied from 86.9% to 94.1%. Each PCR reaction mixture (20 µL final volume) contained 10 µL SYBR Green PCR Master Mix (Applied Biosystems, Warrington, UK), 0.9 µL of 20 µM each primer, 6.2 µL of Milli-Q water, and 2 µL of extracted DNA.

### 2.5. Analytical Procedures

Concentrations of DM (ID 934.01), ash (ID 942.05), and nitrogen (N; ID 984.13) in the diets were determined following the Association of Official Analytical Chemists procedures [38], while those of NDF and ADF were analyzed according to Van Soest et al. [39] using an ANKOM 220 Fibre Analyzer unit (ANKOM Technology Corporation, Fairport, NY, USA). Sodium sulfite and heat-stable amylase were used in the analysis of NDF and ADF, and the results were expressed exclusive of residual ash.

Concentrations of VFA and NH_3_-N in the effluents were determined as described by Martinez et al. [25], and the methane concentration in the gas produced was analyzed by gas chromatography following the procedure of Martinez et al. [40]. Microbial growth was measured using ^15^N as an external microbial marker as proposed by Carro and Miller [29].

### 2.6. Calculations and Statistical Analyses

The efficiency of MPS was determined from the amount of OM apparently fermented, which was estimated from VFA production according to the equation proposed by Demeyer [41]. The diversity of the bacterial communities in the fermenters was determined by Shannon’s diversity index [42]. The relative abundances of fungi and methanogenic archaea were calculated from the absolute quantification of total bacteria as 2 ^− (*CT* target − *CT* total bacteria)^, where *C_T_* represents the threshold cycle after correcting for differences in amplification efficiencies between the target and the reference (total bacteria). Correction factors of the relative qPCR efficiency of archaea and fungi were 1.059 and 1.005, respectively.

Fermentation characteristics and diet disappearance data were analyzed as a mixed model with repeated measures using the PROC MIXED of the Statistical Analysis System (SAS Institute Inc., Cary, NC, USA). The statistical model used included the diet, incubation run, time and diet x time interaction as fixed effects, and fermenter as a random effect. Microbial growth, ARISA, and qPCR data were analyzed as a mixed model using the PROC MIXED of SAS using a model that included diet and incubation run as fixed effects, and fermenter as a random effect. Effects were declared significant at *p* < 0.05, and *p* < 0.10 was considered a trend.

## 3. Results

### 3.1. Diet Disappearance and Rumen Fermentation Parameters

Values of diet disappearance and fermentation parameters for EM and DCP diets are shown in Table 3. The disappearance of NDF and ADF was greater (*p* < 0.001) for DCP than for the EM diet, but there were no differences (*p* > 0.05) between diets on DM and OM disappearance. Fermenters fed the DCP diet showed greater (*p* < 0.001) pH values than those fed the EM diet. Total VFA and NH_3_-N daily productions were unaffected by the diet (*p* > 0.05), but the VFA profile showed differences between EM and DCP fermenters. Replacing EM by DCP resulted in greater (*p ≤* 0.01) propionate, butyrate and valerate proportions and a trend (*p* = 0.06) to increased isobutyrate proportions, as well as lower (*p* ≤ 0.04) acetate, isovalerate, and caproate proportions. As a consequence, the acetate/propionate ratio was lower (*p* < 0.001) for DCP compared with EM fermenters. Fermenters fed the DCP diet had lower (*p* = 0.03) daily methane production and methane/total VFA ratio than those fed the EM diet.

### 3.2. Microbial Protein Synthesis (MPS)

Table 4 shows the MPS and its efficiency, expressed as g of microbial N per kg of OM apparently fermented, in the fermenters. Diet had no effect (*p ≥* 0.14) on either total MPS or the MPS in each digesta phase (SOL and LIQ). Similarly, there were no differences (*p* = 0.91) in the efficiency of MPS between EM and DCP fermenters.

### 3.3. Bacterial Diversity and Microbial Populations

As shown in Table 5, neither the number of peaks nor the Shannon index in the SOL and LIQ phases of fermenters were affected by the diet (*p ≥* 0.46). Diet had no effect (*p* > 0.05) either on the quantity of bacteria and protozoa DNA or on the relative abundance of archaea in the SOL phase of fermenters, but the relative abundance of fungi was greater (*p* < 0.001) in DCP-fed fermenters compared with those fed the EM diet. In the LIQ phase, there was a trend to greater bacteria and protozoa DNA amounts (*p* = 0.098 and 0.06, respectively) and a greater (*p* = 0.02) relative abundance of archaea in DCP-fed fermenters than in EM ones, without differences (*p* = 0.17) in the relative abundance of fungi.

**Figure 1:**
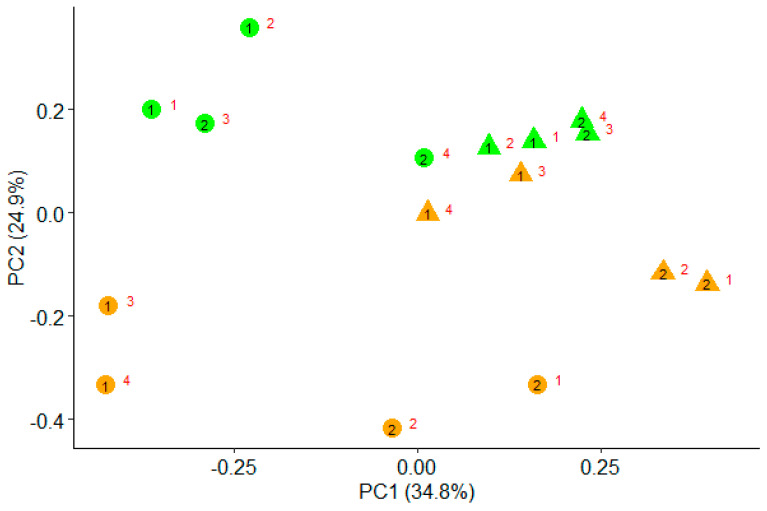
Principal coordinate analysis (PCoA) based on the Bray–Curtis dissimilarities of the automated ribosomal intergenic spacer analysis (ARISA) profiles from liquid (circles) and solid (triangles) phase of fermenters fed a diet containing either extruded maize (green) or dried citrus pulp (orange). Black numbers (1 and 2) correspond to incubation run, and red numbers (1, 2, 3, and 4) to an individual Rusitec fermenter.

Figure 1 represents the PCoA plot based on the Bray–Curtis dissimilarities. Percentages of variance explained by the principal coordinates 1 and 2 were 34.8% and 24.9%, respectively. The plot shows that coordinate 2 separated the samples according to the diet, whereas coordinate 1 separated them according to the digesta phase. Samples from DCP-fed fermenters showed greater separation among them than those fed the EM diet. Regardless of the diet, samples from the SOL phase were placed closer together than those from the LIQ phase.

## 4. Discussion

Although many studies have investigated the effects of DCP on ruminal fermentation, results have been sometimes contradictory, which can be explained by factors such as the type of citrus pulp, the level of dietary inclusion, the ruminant species, the type of diet, and the conventional feed ingredient that were replaced. The greater NDF and ADF disappearance observed for the DCP diet in the present study is in accordance with previous in vivo [15,18,20] and in vitro [43,44] studies, and agrees with the greater cellulolytic bacteria populations observed by Barrios-Urdaneta et al. [20] in the rumen of sheep when DCP replaced barley grains in the diet. Even though results from different studies are contrasting, in most of them, no negative effects of DCP on diet digestibility were reported [19,20,21].

The increased NDF and ADF digestibility of the DCP diet in our study might be also related to the greater pH values observed in the DCP-fed fermenters (6.42), which was more adequate for the fibrolytic activity [45,46] than that in the EM fermenters (6.10). The greater pH promoted by the DCP diet is in accordance to results from studies in which barley grains were replaced by DCP in the diet of sheep [18] and kids [19]. The increased pH values were attributed to the high pectin content of DCP [5,47], whose fermentation in the rumen generates little lactic acid [21,48] and results in lower reductions of pH values compared to fermentation of maize starch. In contrast, no pH changes caused by the inclusion of DCP in the diet have been observed by others. Thus, Zhao et al. [49] reported no differences in rumen pH of Rusitec fermenters fed 50:50 forage:concentrate diets containing either 15% of pure citrus pectin or 15% of pure maize starch. Similarly, no differences in pH were reported when steers fed a tropical grass based diet were supplemented daily with 0, 1.5, or 2.5 kg of pelleted DCP [50], when DCP replaced maize in the concentrate fed to grazing cows [51], or when barley grains were totally replaced by DCP in a 50:50 forage:concentrate diet for dairy sheep [20]. Altogether, these results might indicate that the influence of DCP on rumen pH is more pronounced for high-concentrate than for medium- and high-forage diets. This is consistent with the fact that ruminants fed high-forage diets spend more time ruminating than those fed high-concentrate diets, resulting in greater saliva production and rumen pH. Under these conditions, it would be more difficult to observe the effects of a single feed ingredient on ruminal pH. Despite that Rusitec fermenters are strongly buffered by the continuously infused artificial saliva, our results clearly show that the replacement of extruded maize by DCP in the diet increased rumen pH and maintained them in an adequate values range for fibrolytic activity.

In agreement with previous in vitro [14,21] and in vivo [10,20,23] studies, the total VFA daily production was unaffected by the inclusion of DCP in the diet, but the VFA profile was markedly affected. Studies investigating the influence of DCP on the rumen VFA profile have produced contrasting results, but some of them reported shifts to greater acetate and lower propionate proportions, resulting in greater acetate/propionate ratios [10,21,22]. In contrast, we observed an increase in propionate and a reduction in acetate proportions by including DCP in the diet. In agreement with Oltramari et al. [23], who observed increased butyrate proportions when DCP replaced maize in the starter concentrate of milk-fed dairy calves, the replacement of extruded maize by DCP caused an increase in butyrate proportions in our study. However, no changes in butyrate proportions were detected when DCP replaced either hominy feed in continuous-culture fermenters [21] or barley grains in the diet of dairy ewes [20]. As already discussed, discrepancies in the results from different studies could be due to the composition of both the citrus pulp and the feeds replaced, and to the level of citrus pulp in the diet.

There is little information on the effects of citrus pulp on methane production. Contrary to our results, Homem Junior et al. [52] observed greater in vitro methane production (per gram of incubated DM) when DCP replaced maize grains in the substrate of batch cultures, and Zhao et al. [49] obtained similar results when 15% of pure starch in the diet fed to Rusitec fermenters was substituted by 15% pure citrus pectin. Citrus pulp can contain bioactive compounds, such as terpenoids, limonene, γ-terpinene, citral, or linalool, with antimicrobial activity [53] that might have specific effects on certain ruminal microorganisms. The presence and concentrations of these compounds in citrus pulp are highly variable [9], and their potential effects on methane production deserves further research.

Daily NH_3_-N production was unaffected by the diet, and NH_3_-N concentration was in all fermenters greater than that considered minimum for optimal ruminal digestion [54] and MPS in the rumen [55]. Contrary to our results, several in vitro and in vivo studies reported decreases in NH_3_-N concentrations when the cereals in the diet were partly substituted by DCP. Zhao et al. [49] observed that replacing 15% of starch by 15% pure citrus pectin in a mixed diet fed to Rusitec fermenters caused a reduction in the daily production of NH_3_-N, and Ariza et al. [21] reported similar results in continuous-culture fermenters when DCP replaced dietary hominy feed. A reduction in NH_3_-N concentrations were also observed in the rumen of sheep when DCP substituted either 66% or 100% of barley grains [20] and when ensiled citrus pulp replaced 66% of wheat grains in the diet of dairy sheep [10]. The discrepancies observed among studies can be due to differences in the CP degradability of the feed ingredient replaced by the DCP, but also to differences in microbial growth, as NH_3_-N concentrations reflect the balance between the NH_3_-N produced by CP degradation and that captured by ruminal microorganisms for MPS. Neither MPS nor its efficiency were affected by diet in the present study, which is in agreement with the results of Ariza et al. [21] when hominy feed was replaced by DCP in continuous-culture fermenters, although they observed a trend (*p* = 0.09) to decreased MPS efficiency by the inclusion of DCP. In contrast, Zhao et al. [49] observed an increase in MPS in Rusitec fermenters when 15% of pure starch in the diet was substituted by 15% of pure citrus pectin, but the efficiency of MPS remained unchanged.

The replacement of extruded maize by DCP had no effect on bacterial diversity, but the PCoA plot showed differences in the structure of bacterial populations caused by the diet. Barrios-Urdaneta et al. [20] observed a linear decrease in the concentration of cellulolytic bacteria in the rumen of sheep when increasing proportions of DCP replaced barley grains in the diet, with no changes in amylolytic bacteria concentrations. In contrast, Zhao et al. [49] observed an increase in the 16S rDNA gene copy numbers of *Ruminococcus albus* and *Ruminococcus flavefaciens* in Rusitec fermenters when 15% of pure starch in the diet was replaced by 15% pure citrus pectin. According to Ben-Ghedalia et al. [18], highly digestible fiber, such as that of citrus pulp, provides an excellent substrate for bacterial colonization and increases the available substrates for cellulolytic bacteria. The differences in the structure of the bacterial populations observed in our study could be related with the activity of bioactive compounds naturally present in the citrus pulp, but also with the differences in chemical composition between EM and DCP diets.

Results of qPCR analyses revealed a lack of clear effect of the diet on the quantity of bacteria and protozoa DNA. The numbers of protozoa are drastically reduced after some days of incubation in Rusitec fermenters [56,57] due to the higher outflow rate of protozoa from the fermenters compared to their generation rate, and the low quantity of protozoa DNA observed in the present study is in agreement with previous reports [57]. The greater relative abundance of fungi in the SOL phase of DCP-fed fermenters compared to EM ones is in accordance with the greater NDF disappearance and increased pH values observed in the DCP-fed fermenters, as fungi actively contribute to fiber degradation in the rumen [58] and they are negatively affected by a decrease in pH [59]. The greater relative abundance of methanogenic archaea in the LIQ phase of DCP-fed fermenters contrasts with the lower methane production observed in these fermenters compared with EM ones. However, several studies [60,61] have reported either none or a weak correlation between the numbers of methanogens and methane emissions in ruminants, and a review on this topic [62] reached the same conclusion.

Finally, it is worth mentioning that the results of the PCoA confirmed previous observations [55,63] indicating lower variability in bacterial populations among individual Rusitec fermenters in the SOL compared with the LIQ phase.

## 5. Conclusions

The results indicate that 20% of DCP can be included in a concentrate for dairy sheep replacing the same amount of extruded maize without compromising rumen fermentation and microbial protein synthesis. The inclusion of DCP in the diet only caused subtle changes in microbial populations, but maintained pH values favorable for fibrolytic activity and decreased methane production. Using DCP instead of extruded maize in ruminant diets would reduce the use of potentially human-edible ingredients in animal feeding.

## Figures and Tables

**Table 1:** Ingredient and chemical composition of diet fed to ruminal content donor sheep.

	Diet
**Ingredients (g/kg DM ^1^)**	
Alfalfa hay	300
Maize silage	240
Maize	275
Soybean meal	125
Cottonseed meal	42
Mineral/vitamin premix ^2^	10
Calcium soap of fatty acids	8
**Chemical composition (g/kg DM ^1^)**	
Organic matter	935
Crude protein	186
Neutral detergent fiber	394
Acid detergent fiber	179

^1^ DM: dry matter. ^2^ Declared composition (g/kg mineral/vitamin premix): Vitamin A, 600,000 IU; Vitamin D3, 120,000 IU; Vitamin E, 1 g; Vitamin B1, 33 mg; Niacine, 1.5 g; S, 5 g; IK, 300 mg; SO_4_Fe, 1 g; ZnO, 4 g; MnO, 2 g; CoSO_4_, 60 mg; Na_2_SeO_3_, 30 mg; Ethoxyquin, 30 mg. ^2^ Expressed exclusive of residual ash.

**Table 2:** Ingredient and chemical composition of the experimental diets incubated in the Rusitec fermenters.

	Diet	
	EM ^1^	DCP ^2^
**Ingredients (g/kg DM ^3^)**		
Alfalfa hay	500	500
Barley	123	116
Dried citrus pulp	-	200
Extruded maize	200	-
Soybean meal	158	165
Mineral/vitamin premix ^4^	10	10
Calcium soap of fatty acids	9	9
**Chemical composition (g/kg DM ^3^)**		
Organic matter	944	900
Crude protein	160	160
Neutral detergent fiber ^2^	301	344
Acid detergent fiber ^2^	158	186

^1^ EM: diet containing 20% extruded maize. ^2^ DCP: diet containing 20% dried citrus pulp. ^3^ DM: dry matter. ^4^ Declared composition (g/kg mineral/vitamin premix): Vitamin A, 600,000 IU; Vitamin D3, 120,000 IU; Vitamin E, 1 g; Vitamin B1, 33 mg; Niacine, 1.5 g; S, 5 g; IK, 300 mg; SO_4_Fe, 1 g; ZnO, 4 g; MnO, 2 g; CoSO_4_, 60 mg; Na_2_SeO_3_, 30 mg; Ethoxyquin, 30 mg. ^2^ Expressed exclusive of residual ash.

**Table 3:** Effects of replacing extruded maize (EM) by dried citrus pulp (DCP) on diet disappearance and ruminal fermentation parameters in Rusitec fermenters (n = 4).

Item	Diet		SEM ^1^	*p*-Value
	EM	DCP		
Diet disappearance (g/g)				
Dry matter	0.726	0.740	0.0136	0.11
Organic matter	0.717	0.726	0.0112	0.31
Neutral detergent fiber	0.342	0.426	0.0130	<0.001
Acid detergent fiber	0.200	0.352	0.0174	<0.001
pH	6.10	6.42	0.067	<0.001
NH_3_-N ^2^ (mg/d)	163	194	11.1	0.11
Total VFA ^3^ (mmol/d)	91	89	4.5	0.41
Molar proportions (mol/100 mol)				
Acetate	47.3	46.6	0.45	0.04
Propionate	17.0	19.5	0.55	<0.001
Butyrate	19.8	21.2	0.43	<0.001
Isobutyrate	0.70	0.91	0.152	0.06
Isovalerate	2.75	2.07	0.159	<0.001
Valerate	6.06	6.53	0.186	0.01
Caproate	6.64	3.27	0.278	<0.001
Acetate/propionate (mol/mol)	2.80	2.40	0.091	<0.001
Methane (mmol/d)	25.1	23.3	1.09	0.03
Methane/Total VFA (mol/mol)	0.269	0.262	0.0036	0.03

^1^ SEM: standard error of the mean. ^2^ NH_3_-N: ammonia nitrogen. ^3^ VFA: volatile fatty acids.

**Table 4:** Effects of replacing extruded maize (EM) by dried citrus pulp (DCP) in the diet on microbial protein synthesis in solid (SOL) and liquid (LIQ) phases and its efficiency in Rusitec fermenters (n = 4).

Item	Diet		SEM ^1^	*p*-Value
	EM	DCP		
Microbial protein synthesis (mg N/d)				
SOL	180	200	7.7	0.14
LIQ	137	116	7.9	0.14
Total	317	316	6.5	0.92
Efficiency of microbial growth ^2^	34.9	34.7	1.46	0.91

^1^ SEM: standard error of the mean. ^2^ Expressed as mg N per g organic matter apparently fermented.

**Table 5:** Effects of replacing extruded maize (EM) by dried citrus pulp (DCP) in the diet on the number of peaks and Shannon diversity index analyzed by automated ribosomal intergenic spacer analysis (ARISA), the abundance of bacteria and protozoa DNA, and the relative abundance of fungi and archaea determined by qPCR in solid (SOL) and liquid (LIQ) phases of Rusitec fermenters (n = 4).

Phase	Item	Diet		SEM ^1^	*p*-Value
		EM	DCP		
**SOL**	**Bacterial diversity (ARISA)**				
	Number of peaks	28.5	27.5	0.64	0.70
	Shannon index	3.35	3.31	0.044	0.66
	**Microbial populations (qPCR)**				
	Total bacteria ^2^	131	118	14.8	0.77
	Total protozoa ^2^	0.0014	0.0018	0.00017	0.45
	Fungi ^3^	0.004	8.799	0.5429	<0.001
	Archaea ^3^	0.030	0.032	0.0020	0.77
**LIQ**	**Bacterial diversity (ARISA)**				
	Number of peaks	30.8	34.8	1.73	0.57
	Shannon index	3.35	3.54	0.068	0.46
	**Microbial populations (qPCR)**				
	Total bacteria ^2^	1.12	2.91	0.443	0.098
	Total protozoa ^2^	0.0001	0.0004	0.00004	0.06
	Fungi ^3^	0.004	0.039	0.0073	0.17
	Archaea ^3^	0.007	1.739	0.1426	0.02

^1^ SEM: standard error of the mean. ^2^ Expressed as µg DNA/g DM for SOL and µg DNA/mL for LIQ. ^3^ Expressed as relative abundance to the absolute quantification of total bacteria as 2 ^− (*CT* target *− CT* total bacteria)^.
